# Ekbom Syndrome associated with Lewy Body Dementia: A case report

**DOI:** 10.1590/1980-57642020dn14-010014

**Published:** 2020

**Authors:** Felippe José Pontes de Mendonça, Ivan Abdalla Teixeira, Valeska Marinho

**Affiliations:** 1 Center for Alzheimer’s Disease and Related Disorders, Institute of Psychiatry – Universidade Federal do Rio de Janeiro, RJ, Brazil

**Keywords:** Ekbom syndrome, parasitosis delusion, psychogenic parasitosis, acarophobia, delusional disorders, self-harm, dementias, Lewy Body Dementia, síndrome de Ekbom, delírio parasitário, parasitose psicogênica, acarofobia, transtornos delirantes, automutilação, demências, demência com corpos de Lewy

## Abstract

Ekbom Syndrome, also known as parasitosis delusion or psychogenic parasitosis, is a rare condition in which patients present with a fixed belief of being infested by parasites, vermin or small insects, along with tactile hallucinations (such as pruritus or sensations of the parasites crawling over or under the skin). The syndrome may occur idiopathically or be associated with other medical conditions and drug use. This case report describes the occurrence of Ekbom syndrome in a patient diagnosed with Lewy Body Dementia (LBD), a neurodegenerative disease that commonly presents with sensory perception and thought disorders and other neuropsychiatric symptoms. Although visual hallucination is considered a core diagnostic criterion, other modalities of psychiatric symptoms can also occur posing a further challenge for correct diagnosis. Proper recognition allows early diagnosis and adequate treatment, preventing hazardous antipsychotic use in these patients.

## RESUMO

A síndrome de Ekbom, também conhecida como delírio parasitário ou parasitose psicogênica, é uma condição rara na qual os pacientes apresentam crença fixa de estarem infestados por parasitas, vermes ou insetos, acompanhada de alucinações táteis (como prurido ou sensação dos parasitas andando sobre ou sob a pele). A síndrome pode ocorrer de forma idiopática ou associada a outras condições médicas ou uso de drogas. Este relato de caso descreve a ocorrência da síndrome de Ekbom em um paciente diagnosticado com Demência com corpos de Lewy (DCL), uma doença degenerativa que comumente se apresenta com desordens de sensopercepção e pensamento, e outros sintomas neuropsiquiátricos. A alucinação visual é considerada um dos critérios diagnósticos nucleares, entretanto outras modalidades de sintomas psiquiátricos podem ocorrer criando desafios adicionais ao diagnóstico correto. O reconhecimento apropriado permite o diagnóstico precoce e tratamento adequado, prevenindo o uso arriscado de antipsicóticos nesses pacientes.

Ekbom syndrome manifests as a delusion of infestation of the body by parasites or insects,^1^ where corresponding tactile hallucinations may occur together with the delusional disorder.^2^ A variety of medical conditions may be associated with this syndrome: hypothyroidism, neurodegenerative diseases, anemia, diabetes, peripheral neuropathies, vitamin deficiencies, or drug abuse.^3^ Although there is currently no consensus on the underlying pathophysiology, lesions involving the temporal lobes, subcortical circuits^4^ and limbic areas^5^ are frequently associated.

The syndrome is most common in women^8^ in the fifth decade of life, with an estimated prevalence of 83.21 million people.^9^ Typically, the symptoms lead to self-mutilation in attempts to remove the parasites, causing excoriations and scarring.^9^ The most affected areas are the scalp, face, mouth, eyes, arms, breasts and genital regions.^9^ Risk factors include isolation, poor social support and low educational status.^10^

Lewy Body Dementia (LBD) is the second-most-frequent cause of degenerative dementia.^11^ LBD core features are fluctuating cognition, Rapid Eye Movement (REM) behavior disorder, visual hallucinations and parkinsonism. Among the neuropsychiatric symptoms, great emphasis is placed on sensory perceptual disorders, especially visual hallucinations.^12^^,^ 13 However, psychiatric manifestations in LBD can be complex and highly heterogenous,^14^ and other symptoms have also been described, such as psychotic depression,^14^^,^ 15 catatonia^16^ and delusional disorders (e.g. Capgras syndrome).^17^

Ekbom syndrome is a rare presentation in LBD and was previously described in two case reports of the disease managed with atypical antipsychotics.^18^ However, severe neuroleptic sensitivity may affect up to 50% of LBD patients and is characterized by worsening cognition, sedation, acute onset parkinsonism and symptoms resembling neuroleptic malignant syndrome.^19^ Owing to these risks, tailored management is advisable to reduce morbidity and mortality due to excessive neuroleptic use.^20^ In this scenario, acetylcholinesterase inhibitors (AChEIs) seem a reasonable choice to treat psychotic symptoms in LBD, but robust evidence for their efficacy in treating these symptoms is still lacking. Considering these issues, we report an LBD case, first presenting as Ekbom syndrome, successfully managed with AchEI, preventing potentially harmful antipsychotic use.

## CASE REPORT

We describe a 72-year-old male retired taxi driver, presenting with complaints of “bugs in his head. Previous medical history included hypertension, dyslipidemia, hypothyroidism and benign prostatic hyperplasia, with no personal or family history of neuropsychiatric disorders.

At 70 years old, he started having trouble finding his passengers addresses, handling money and using smartphone apps, necessary for his work. He caused a car crash when trying to use his smartphone while driving and, a few days later, damaged two tyres while parking. After this, his family persuaded him to stop working, which made him feel increasingly sad and “useless”.

Two years later, he started complaining of “ants” crawling on his head. He started scratching and picking it, claiming that an “ants nest” had infested his scalp. This belief firmly persisted despite being contradicted by his family and given the logical arguments against it. He was restless at night, with excessive limb movements and talking while asleep. He started seeing small non-existent animals, like spiders, frequently. In addition, he was also hearing sounds inaudible to others such as whistling and buzzing sounds, which he sometimes attributed to the “bugs”, besides hearing his name being called or the doorbell ringing several times a day. Since giving up work, he had persistent depressive symptoms, which included loss of interest in previously pleasurable activities and interactions with his family, persistent low mood and sobbing, self-deprecating ideas such as recurring thoughts of feeling useless and a burden to his family, low energy, apathy and hyporexia.

He remained independent for basic activities of daily living, but was already dependent for instrumental activities, such as cooking and financial management. Upon arrival at our service, he already displayed initial memory deficits, repeating himself in conversations and forgetting recent events.

At neurological examination, he was lucid with hypomimia and hypophonia, presenting unsteady gait with reduced arm movements, mild postural instability on the pull test, moderate bilateral bradykinesia in upper and lower limbs, mild cogwheel rigidity, no resting or kinetic tremor, with preserved muscle strength and no disturbances in sensitivity. MDS-UPDRS^21^ total score was 64. Psychiatric evaluation detected apathy and passivity, overall low mood, few expressions of emotion, mostly when talking about the “ants” on his head, indicating where he felt them, and expressing great anguish. He also cried while talking about his past performance at work and home compared to his current status, demonstrating insight of his deficits. He had no trouble remembering remote past life events and memory problems were restricted to recent events.

On the initial cognitive assessment, he scored 25/30 points on the MMSE;^22^^,^ 23 9 points on the semantic verbal fluency test^24^ (animals category) and 2/3 points on the clock-drawing test.^26^ He scored 16/30 points on the MoCA^26^^,^ 27 and was classified as CDR 1 on the Clinical Dementia Rating scale.^28^^,^ 29 Extensive neuropsychological testing was performed, revealing immediate and delayed verbal memory recall deficits, moderate to severe executive dysfunction, including difficulties with sequencing, planning, cognitive flexibility, and deficits in abstracting and conceptualizing abilities. He scored 37 points on the Neuropsychiatric Inventory (NPI),^30^^,^ 31 mainly involving the categories: hallucinations, depression, apathy/indifference, sleep and appetite disturbances.

**Figure 1:**
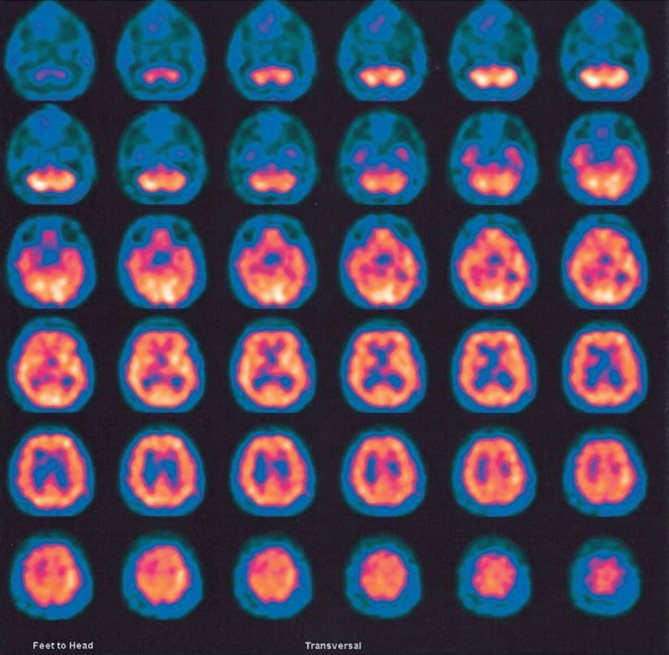
DAT SPECT imaging showing reduced radiotracer uptake in the basal ganglia.

**Figure 2:**
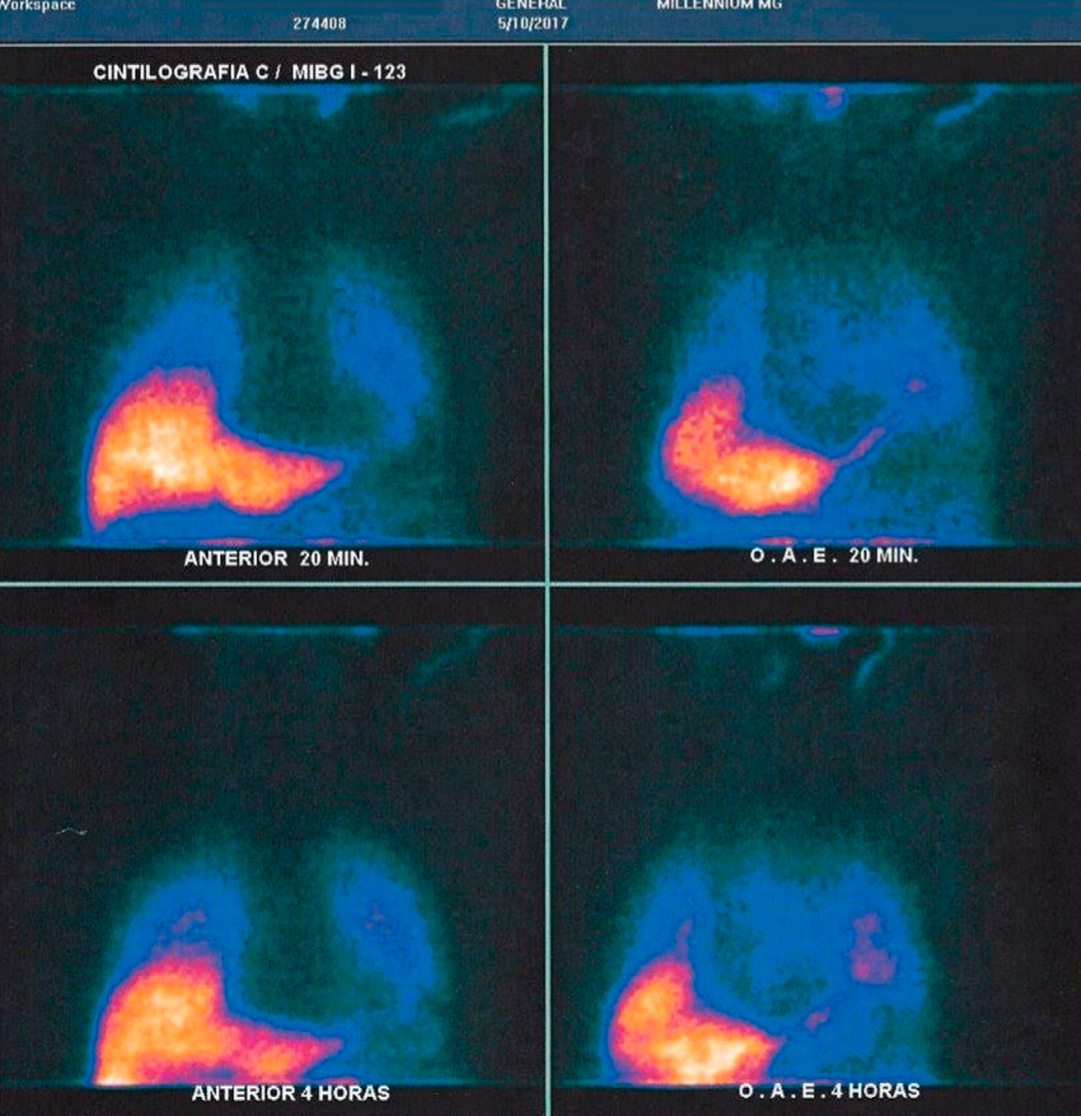
MIBG-123I Scintigraphy showing reduced radiotracer uptake in cardiac chambers, indicating significant sympathetic denervation.

Brain MRI revealed diffuse cortical atrophy and mild microangiopathy predominantlin frontoparietal and periventricular subcortical white matter, and mild bilateral hippocampal reduction, all compatible with the patient’s age and degree of cortical atrophy. Perfusion SPECT study showed no abnormalities; the DAT SPECT imaging, however, revealed reduced radiotracer uptake in the basal ganglia indicating moderate nigro-striatal dysfunction (Figure 1). MIBG-123I Cardiac Scintigraphy was also performed, suggesting significant cardiac sympathetic denervation (Figure 2). Polysomnography was inconclusive, as the patient did not achieve REM sleep. The diagnosis of probable LBD was established based on clinical symptoms and presence of two positive biomarkers.

Depressive symptoms were initially treated with citalopram. Rivastigmine was then started and titrated to maximum daily dose. Most of the hallucinatory and delusional symptoms improved significantly only after starting on AChEI, and Ekbom syndrome disappeared, with no need for antipsychotic medication.

## DISCUSSION

A diagnosis of probable Lewy Body Dementia was established considering the criteria of the latest consensus of experts. Two of the four core clinical features were present: visual hallucinations and spontaneous parkinsonism. There were also symptoms suggestive of REM sleep behavior disorder. It is noteworthy that initial cognitive symptoms were not memory deficits, but attention, executive and visuospatial deficits, as expected in LBD. Supporting clinical features were also present: postural instability; hallucinations in other modalities (auditory, tactile); delusions of insect infestations; depression. LBD is known for its tendency to cause sensory and perceptual disturbances early in the disease course. Tactile hallucinations were part of the Ekbom syndrome presented by the patient, leading to self-harm. Two of the indicative biomarkers were positive: the DAT SPECT imaging and MIBG Myocardial scintigraphy. A supporting biomarker – the structural MRI showing preservation of medial temporal structures – was helpful to exclude Alzheimer’s disease. Moreover, motor symptoms only surfaced two years after the onset of cognitive symptoms, excluding the diagnosis of Parkinson’s disease.

Management of psychotic symptoms in LBD is challenging, given the uncertain benefits of antipsychotic use, together with the risks of serious adverse events. A few studies, with less rigorous methodology in LBD and Parkinson’s Disease Dementia, have failed to demonstrate evidence of efficacy of antipsychotics in these conditions. Expert consensus suggests that clozapine and quetiapine may be considered as options to control distressing behavioral symptoms in LBD, but careful monitoring for clozapine, lack of evidence for quetiapine, severe neuroleptic sensitivity and overall increased mortality constitute additional concerns inherent to these choices.^32^ In this scenario, AChEIs represent plausible options. In a recent systematic review of pharmacological strategies in LBD, AChEIs demonstrated good evidence for controlling cognitive and behavioral symptoms, suggesting that these medications can be a reasonable first choice to treat psychotic symptoms.^32^

As illustrated here, there is a need for increasing awareness of late onset psychiatric symptoms as a dementia prodrome. Proper diagnosis can guide disease management and provide maximum benefit, while minimizing adverse effects, the mainstay of good medical practice.
